# Evaluation of neuroendocrine markers in renal cell carcinoma

**DOI:** 10.1186/1746-1596-5-28

**Published:** 2010-05-12

**Authors:** Hanna Ronkainen, Ylermi Soini, Markku H Vaarala, Saila Kauppila, Pasi Hirvikoski

**Affiliations:** 1 Department of Surgery, PO Box 21, Oulu University Hospital, FIN-90029 Oulu, Finland; 2 Department of Clinical Pathology, PO Box 1777, Kuopio University Hospital, FIN-70211 Kuopio, Finland; 3 Department of Pathology, PO Box 50, Oulu University Hospital and University of Oulu, FIN-90029 Oulu, Finland

## Abstract

**Background:**

The purpose of the study was to examine serotonin, CD56, neurone-specific enolase (NSE), chromogranin A and synaptophysin by immunohistochemistry in renal cell carcinomas (RCCs) with special emphasis on patient outcome.

**Methods:**

We studied 152 patients with primary RCCs who underwent surgery for the removal of kidney tumours between 1990 and 1999. The mean follow-up was 90 months. The expression of neuroendocrine (NE) markers was determined by immunohistochemical staining using commercially available monoclonal antibodies. Results were correlated with patient age, clinical stage, Fuhrman grade and patient outcome.

**Results:**

Eight percent of tumours were positive for serotonin, 18% for CD56 and 48% for NSE. Chromogranin A immunostaining was negative and only 1% of the tumours were synaptophysin immunopositive. The NSE immunopositivity was more common in clear cell RCCs than in other subtypes (*p *= 0.01). The other NE markers did not show any association with the histological subtype. Tumours with an immunopositivity for serotonin had a longer RCC-specific survival and tumours with an immunopositivity for CD56 and NSE had a shorter RCC-specific survival but the difference was not significant. There was no relationship between stage or Fuhrman grade and immunoreactivity for serotonin, CD56 and NSE.

**Conclusions:**

Serotonin, CD56 and NSE but not synaptophysin and chromogranin A are expressed in RCCs. However, the prognostic potential of these markers remains obscure.

## Background

Neuroendocrine (NE) cells are important for regulating cell growth and differentiation. In addition to specific NE tumours, NE activity can be detected in other types of tumours such as breast [1] or prostate carcinomas [2]. The specific NE tumours of kidney include carcinoid, NE carcinoma, primitive neuroectodermal tumour, neuroblastoma and phaeochromocytoma [3]. NE tumours can show a wide range of behaviour. Small cell carcinomas of the lung are aggressive [4], whereas carcinoid tumours show indolent behaviour [5]. In patients with prostate adenocarcinoma, NE differentiation has been linked to both aggressive behaviour [6] and better survival [7].

Serotonin (5-hydroxytryptamine, 5HT) is a growth factor for several types of malignant cells. Serotonin causes cellular proliferation [8], and there is also evidence linking it to oncogenes [9]. By contrast, serotonin can also inhibit tumour growth because of its vasoconstrictive effect [10]. In RCC patients, plasma levels of serotonin [11] and the immunoexpression for serotonin have previously been examined in patients with advanced disease [12]. As far as we know, the prognostic significance of serotonin expression in RCC patients has not been studied in large RCC patient material.

CD56 is neural cell adhesion molecule (NCAM), which is also found in some lymphocytes [13]. In terms of clinical pathology, CD56 is a rather sensitive indicator of NE differentiation. The immunoexpression of CD56 has previously been studied in RCC and its prognostic potential in the survival of RCC patients has also been evaluated [14].

Neurone-specific enolase (NSE) is a broad-spectrum, non-specific NE marker of all types of neurons, NE or paraneuronal cells and even various malignant tumours of non-NE types [15]. Its serum levels and immunoexpression have also been studied in RCCs [16,17,12].

Chromogranin A is an abundant monomeric protein in the neurosecretory granules of NE cells, and its immunostaining correlates to the number of NE granules seen at the level of electron microscopy [13]. The serum levels and immunoexpression of chromogranin A have previously been studied in RCCs to evaluate its prognostic significance [12,17].

Synaptophysin is regarded as one of the basic markers of NE differentiation. It is an integral part of the NE secretory granule membrane [13]. To our knowledge, the immunoexpression of synaptophysin has not previously been studied in RCCs.

The aim of this study was to clarify the extent of the immunoexpression of NE markers, serotonin, CD56, NSE, chromogranin A and synaptophysin in RCCs and their significance regarding the behaviour of these tumours. For this we investigated a large set of RCCs consisting of different histological types and correlated the results with the clinical behaviour of the tumours.

## Methods

The retrospective study group consisted of 152 patients treated with radical nephrectomy or renal resection for primary RCC at Oulu University Hospital, Oulu, Finland between 1990 and 1999. Patients underwent medical examination and preoperative staging including chest X-ray and/or thoracic CT and abdominal CT. The research plan was approved by the local ethics board. All the data from the patients' records and Finnish Cancer Registry were re-evaluated by the same urologist. The exact stage of the disease was recorded according to the TNM classification of RCCs [18].

Archival material of formalin-fixed and paraffin-embedded tumours were reclassified and graded according to current WHO classification [3]. The most representative area from each tumour block was selected to a multitissue array block. The array section was 3 μm thick.

### Immunostaining procedure

The antibodies used in the immunostaining were monoclonal mouse anti-human serotonin (DakoCytomation, Glostrup, Denmark) in a dilution of 1:200, lyophilized mouse monoclonal antibody for CD56 (Novocastra Laboratories Ltd., Newcastle-upon-Tyne, UK) in a dilution of 1:200, monoclonal mouse anti-NSE (Zymed Laboratories, Carlsbad, CA, USA) in a dilution 1:1000, polyclonal rabbit anti-chromogranin A (Zymed Laboratories, Carlsbad, CA, USA) in a dilution 1:500 and monoclonal mouse anti-synaptophysin (DakoCytomation) in a dilution 1:50. First, the sections were deparaffinised in xylene, rehydrated in descending ethanol series and washed in phosphate-buffered saline (PBS). Then, the sections were boiled in 0.01 M citrate buffer (pH 6) for 10 min (serotonin and CD56) or Tris/EDTA for 15 min (chromogranin A and synaptophysin) in a microwave oven. The sections were cooled for 15 min and washed twice in PBS. Endogenous peroxidise activity was eliminated by incubation in 5% hydrogen peroxide and absolute methanol. Bound antibodies were visualised using an UltraVision (Thermo Fisher Scientific, Fremont, CA, USA) for serotonin, CD56 and NSE and EnVision (DakoCytomation) for chromogranin A and synaptophysin. DAB (5% 3,3'-diaminobenzidine tetrahydrochloride, DakoCytomation) was used as the chromogen. The positive controls for stainings were the metastasis of neuroendocrine tumour for serotonin, small cell carcinoma for CD56 and NSE, and colonic mucosa for chromogranin A and synaptophysin. PBS instead of primary antibody was used as a negative control.

### Immunohistochemical evaluation of NE markers

Cytoplasmic immunostaining for serotonin, CD56, NSE, chromogranin A and synaptophysin was classified dichotomously as positive or negative simultaneously by three observers (HR, PH and SK).

### Statistical analyses

SPSS for Windows 15 (Chicago, IL, USA) was used for statistical analyses. Statistical significance between stainings and clinicopathological parameters was determined using the chi-squared test or Fisher's exact test in the case of low expected frequencies. Corrected cancer-specific survival was analysed with the Kaplan-Meier curve and the significance with the log rank test. The Cox regression model was used for multivariate analysis.

## Results

### Patients, follow-up and treatment

The median age of the patients was 63 (range 29-86) years. Seventy-seven (51%) patients were women, 75 (49%) men. Seven tumours (5%) were resected and 145 (95%) were operated by radical nephrectomy. The median follow-up time was 90 (range 0-209) months and follow-up was complete in all cases. During follow-up, 44 (29%) patients died of RCC, 40 (26%) died of other causes and 68 (45%) patients were still alive. The distribution of clinicopathological parameters of the tumours is described in Table 1.

**Table 1:** Distribution of clinical stages and T classes, nuclear grade and histological evaluation.

	n (%)
Clinical stage	
I	70 (46)
II	12 (8)
III	51 (34)
IV	19 (12)
	
Tumour class	
T1	75 (49)
T2	12 (8)
T3	59 (39)
T4	6 (4)
	
Nuclear grade	
grade I	5 (3)
grade II	83 (55)
grade III	40 (27)
grade IV	22 (15)
	
Histological subtype	
clear cell	134 (88.2)
papillary	11 (7.2)
chromophobic	5 (3.3)
unclassified	2 (1.3)

### Immunohistochemical findings

Twelve tumours (8%) were stained for serotonin and 26 (18%) for CD56. The immunopositivity for NSE was detected in 69 cases (48%). The immunopositivity for serotonin and CD56 were detected in the same tumours (p < 0.001). All tumours were immunonegative for chromogranin A (100%). Two of the tumours (1%) were immunopositive for synaptophysin.

The immunopositivity for NSE was more common in clear cell RCCs than other subtypes (*p *= 0.01). There was no association between the immunoreactivity for other NE markers and histological subtype of the tumours (Table 2).

**Table 2:** Association between immunoreactivity for NE markers and histological subtype of RCC.

		Immunoreactivity		
Histological subtype	Marker	negative, n (%)	positive, n (%)	p-value
clear cell	serotonin	121 (93)	9 (7)	0.2
papillary		6 (75)	2 (25)	
chromophobic		4 (80)	1 (20)	
unclassified		2 (100)	0 (0)	
				
clear cell	CD56	105 (82)	23 (18)	0.9
papillary		7 (78)	2 (22)	
chromophobic		4 (80)	1 (20)	
unclassified		2 (100)	0 (0)	
				
clear cell	NSE	62 (48)	67 (52)	0.01
papillary		8 (80)	2 (20)	
chromophobic		4 (100)	0 (0)	
unclassified		2 (100)	0 (0)	
				
clear cell	synaptophysin	130 (99)	1 (1)	0.2
papillary		9 (90)	1 (10)	
chromophobic		5 (100)	0 (0)	
unclassified		2 (100)	0 (0)	

**Figure 1:**
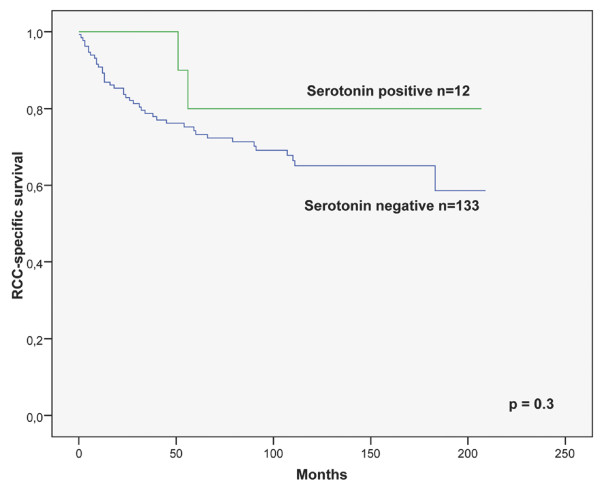
**Cytoplasmic serotonin as a prognostic factor in RCC-specific survival**. Kaplan-Meier curve of 145 patients (p = 0.3).

The RCC-specific survival of patients with serotonin positive tumours (Figure 1) as well as CD56 and NSE immunonegative tumours was somewhat better but the difference was not significant (Table 3). The two patients with synaptophysin immunopositive tumours showed an excellent RCC-specific survival.

**Table 3:** RCC-specific mean survivals for serotonin, CD56 and NSE.

Marker	Immunoreactivity	Survival, months (95% CI)	p-value
serotonin	positive	176 (138-214)	0.3
	negative	148 (133-163)	
CD56	positive	139 (106-172)	0.4
	negative	156 (140-172)	
NSE	positive	149 (127-171)	0.8
	negative	153 (134-171)	

CI, confidence interval.

Immunostaining for serotonin, CD56 and NSE did not associate with grade or stage (Table 4). Each staining (serotonin, CD56 and NSE) was separately included in the Cox multivariate analysis with stage, Fuhrman grade and age. The only significant prognostic factor for RCC-specific survival was stage (*p *< 0.001).

**Table 4:** Association between immunostaining and Fuhrman grade and stage.

		Immunostaining		
Fuhrman grade or stage	Marker	immunonegative, n (%)	immunopositive, n (%)	p-value
grade I	serotonin	5 (100)	0 (0)	0.2
grade II		75 (95)	4 (5)	
grade III		31 (84)	6 (16)	
grade IV		20 (91)	2 (9)	
				
stage I	serotonin	60 (91)	6 (9)	0.6
stage II		9 (90)	1 (10)	
stage III		45 (90)	5 (10)	
stage IV		19 (100)	0 (0)	
				
grade I	CD56	5 (100)	0 (0)	0.7
grade II		65 (83)	13 (17)	
grade III		29 (78)	8 (22)	
grade IV		17 (77)	5 (23)	
				
stage I	CD56	59 (88)	8 (12)	0.2
stage II		7 (70)	3 (30)	
stage III		38 (78)	11 (22)	
stage IV		14 (78)	4 (22)	
				
grade I	NSE	0 (0)	5 (100)	0.1
grade II		41 (52)	38 (48)	
grade III		22 (56)	17 (44)	
grade IV		11 (55)	9 (45)	
				
stage I	NSE	35 (52)	32 (48)	0.6
stage II		6 (55)	5 (45)	
stage III		28 (57)	21 (43)	
stage IV		7 (39)	11 (61)	

## Discussion

Evidence regarding the prognostic influence of NE markers in human carcinomas is confusing. For example, in prostate cancer both the increased and decreased immunoexpression of NE marker serotonin have been associated with advanced or aggressive disease [6,7]. In undifferentiated endometrial carcinomas, NE markers had no prognostic significance [19]. In addition, the clinical behaviour of breast carcinomas with NE differentiation is not well established [20]. The current paper examines the expression of NE markers (serotonin, CD56, chromogranin A, synaptophysin and NSE) in RCC with a special reference for patient survival.

Serotonin is a monoamine neurotransmitter mediating a wide range of physiological actions in the human body. Among others it is implicated in psychiatric and neurological disorders and also plays a fundamental role in tumour growth, differentiation and gene expression [8]. Serotonin has been proposed to take part in the autocrine loops of growth factors, contributing to cell proliferation in aggressive tumours such as small cell lung carcinoma. By contrast, serotonin can inhibit tumour growth, which is thought to be related to its vasoconstrictive effect [10]. It has been shown that decreased serotonin immunoexpression is associated with the progression of prostate cancer [7]. Furthermore, serotonin receptor overexpression is demonstrated in high-grade tumours and serotonin seems to stimulate prostate cancer cells [6]. In RCC, it has been shown that plasma serotonin levels are decreased in patients with metastases but there was no significant association between plasma serotonin level and the extent of the disease [11]. In a previous study of 10 patients with advanced RCC, no immunoexpression of serotonin was detected [12]. In the current study, the immunostaining for serotonin was uncommon, and only 8% of tumours were serotonin immunopositive. The discrepancy in detected immunoexpression with the earlier study [12] might be because of different stage groups and a larger study population. The immunoreactivity for serotonin was detected only in localised or locally advanced disease. None of the tumours with distal metastases were immunopositive for serotonin. However, in terms of patient survival, the immunohistochemical expression of serotonin in RCC seems to have no clinical significance.

CD56 is a member of the immunoglobulin superfamily. Only 15% of conventional RCCs express CD56 and the positivity is associated with poor patient outcome [14]. In addition, the expression of CD56 has been associated with a higher risk of adrenal gland and central nervous system metastases, tumour size, renal vein involvement, perirenal invasion and aggressive Fuhrman grade [14] The current data, however, did not support this observation (data not shown). In our study, there was immunostaining for CD56 in 18% of clear cell RCCs, which is consistent with the earlier study. However, the same frequency of CD56 positivity in papillary and chromophobic RCCs was also detected. The expression of CD56 was not associated with stage or grade. Despite a shorter mean survival for CD56 positive tumours, the expression of CD56 was not a significant predictive marker for RCC in the current study.

NSE is a rather non-specific marker of NE differentiation, which can be found in a variety of normal and neoplastic NE cells as well as in any type of neoplasms even of non-NE origin [15,13]. Elevated serum NSE levels can be detected in 27-80% of patients with RCC [16,17] and seem to be associated with patient outcome [17]. After the treatment of RCC, serum levels of NSE decrease and it has been suggested that this could be a useful marker in the surveillance of RCC [16]. The immunoreactivity for NSE in RCC has been found to be up to 100% [12,17]. In our study, half of tumours were immunopositive for NSE. The immunoreactivity for NSE did not associate with stage or grade. Positivity for NSE was more common in clear cell carcinomas than other subtypes of RCCs. Immunostaining for NSE was not a prognostic factor in RCC-specific survival. In our study population, the expression of NSE was not as common as in previous studies and did not show any prognostic significance in RCC.

Chromogranin A was originally identified as a major soluble protein in adrenal medullary chromaffin granules many decades ago [21]. It has been intensively studied with respect of its physiological role and pathological expression in tumours [22]. In a previous study, serum chromogranin A was found to be elevated in 14% of RCC patients without any prognostic significance in RCC [17]. The immunohistochemical reactivity for chromogranin A has been detected in 4% of RCC patients [17] or the tumours have been wholly immunonegative for chromogranin A [12]. In our study, we found no immunoreactivity for chromogranin A in RCCs, which is consistent with the latter study.

Synaptophysin is a transmembrane glycoprotein of presynaptic vesicles of nerve endings [23,24]. It is regarded as one of the most specific markers of NE differentiation. The immunoexpression of synaptophysin has previously been described in undifferentiated endometrial carcinomas where it did not seem to have a significant prognostic potential [19]. To our knowledge, this is the first study describing the immunostaining of synaptophysin in RCC. In our study, immunostaining for synaptophysin was rare (only 1% of the tumours). Interestingly, the prognosis of patients with synaptophysin immunopositive tumours was excellent. Both patients whose primary RCC tumour showed an immunopositivity for synaptophysin are still alive after 10 and 17 years follow-up. The TNM classes for these synaptophysin immunopositive tumours were pT1aN0M0 and pT3aN0M0. Synaptophysin positivity in RCC may be associated with good prognosis, but as an uncommon phenomenon, this observation should be further studied with large number of RCC tumours.

## Conclusions

To conclude, the immunohistochemical expression of NE markers was quite common in RCC. The current study showed that serotonin, CD56, chromogranin A, synaptophysin and NSE were not potential prognostic markers in RCCs. The outcome of an individual patient with RCC is still to be evaluated with traditional clinicopathological markers such as stage and grade.

## Competing interests

The authors declare that they have no competing interests.

## Authors' contributions

HR, SK and PH evaluated the immunohistochemical staining. HR performed statistical analyses. All authors revised the manuscript and approved the final manuscript.
